# Proof-of-concept for CRISPR/Cas9 gene editing in human preadipocytes: Deletion of FKBP5 and PPARG and effects on adipocyte differentiation and metabolism

**DOI:** 10.1038/s41598-020-67293-y

**Published:** 2020-06-29

**Authors:** Prasad G. Kamble, Susanne Hetty, Milica Vranic, Kristina Almby, Casimiro Castillejo-López, Xesús M. Abalo, Maria J. Pereira, Jan W. Eriksson

**Affiliations:** 1 Department of Medical Sciences, Clinical Diabetology and Metabolism, Uppsala University, Uppsala, Sweden; 2 Department of Immunology, Genetics and Pathology. Science for Life Laboratory, Uppsala University, Uppsala, Sweden

**Keywords:** Biological techniques, Physiology

## Abstract

CRISPR/Cas9 has revolutionized the genome-editing field. So far, successful application in human adipose tissue has not been convincingly shown. We present a method for gene knockout using electroporation in preadipocytes from human adipose tissue that achieved at least 90% efficiency without any need for selection of edited cells or clonal isolation. We knocked out the *FKBP5* and *PPARG* genes in preadipocytes and studied the resulting phenotypes. PPARG knockout prevented differentiation into adipocytes. Conversely, deletion of FKBP51, the protein coded by the *FKBP5* gene, did not affect adipogenesis. Instead, it markedly modulated glucocorticoid effects on adipocyte glucose metabolism and, furthermore, we show some evidence of altered transcriptional activity of glucocorticoid receptors. This has potential implications for the development of insulin resistance and type 2 diabetes. The reported method is simple, easy to adapt, and enables the use of human primary preadipocytes instead of animal adipose cell models to assess the role of key genes and their products in adipose tissue development, metabolism and pathobiology.

## Introduction

Adipose tissue is widely regarded as an endocrine organ that plays a central role in both obesity and insulin resistance^1^. Dysregulation of adipose tissue transcriptional pathways may contribute to significant changes in energy balance, glucose and lipid metabolism, and adipokine secretion, which in turn can influence the whole-body metabolic homeostasis. Thus, identification of genes and functional assessment of their corresponding proteins involved in such pathways could help discover novel disease mechanisms that could be used for drug development. Different approaches such as pharmacological inhibition using receptor antagonists or neutralizing antibodies, as well as genetic manipulation using small interfering RNA-mediated knockdown^2^ have been widely opted for studying the function of different gene products in human adipose cells. However, the specificity and stability of such approaches are critical factors and may sometimes limit their use.

The recent advancements in clustered regularly interspaced short palindromic repeats/CRISPR-associated nuclease 9 (CRISPR/Cas9) technology have given rise to a precise and highly efficient method for gene editing in cells, tissues and whole organisms^3^. However, its application to human adipose tissue is scarce. To the best of our knowledge, only one study reported using this technique in primary human adipose cells, but the phenotypic results following a single nucleotide substitution in the fat mass and obesity-associated gene were equivocal^4^. In another study, immortalized human brown preadipocytes were used to knockout genes using CRISPR/Cas9^5^. Both of these studies have used an expression vector to deliver the CRISPR components into the cells. However, there are some potential complications associated with the use of plasmid DNA, as mentioned in the discussion.

Our laboratory has a long-standing interest in mechanisms by which glucocorticoids cause insulin resistance in human adipose tissue. In a previous microarray study, we identified genes that were differentially regulated by *ex vivo* treatment of human adipose tissue with a synthetic glucocorticoid, dexamethasone^6^. We found that FK506 binding protein 5 (*FKBP5)* was among the genes whose expression was increased the most in response to dexamethasone. Its expression in adipose tissue alone, and in response to dexamethasone, was associated with markers of insulin resistance^6^. Also, variants in the *FKBP5* gene were shown to be associated with type 2 diabetes and diabetes-related phenotypes. The activity of FK506 binding protein 51 (FKBP51), an immune-modulating protein corresponding the *FKBP5* gene, has been extensively studied concerning psychiatric disorders^7,8^, but it has also increasingly emerged as a systemic player in metabolic regulation based on its high expression in metabolically active tissues such as skeletal muscle and adipose tissue^8^. FKBP51 has been considered as a mainly negative regulator of glucocorticoid action^9^ and therefore we studied its role in the context of glucocorticoid effects on adipose tissue metabolism.

In the present study, we aimed to establish the CRISPR/Cas9 method for gene knockout studies in isolated human primary preadipocytes. As a proof-of-concept, we deleted FKBP51 in preadipocytes and investigated its role in adipogenesis and in the context of glucocorticoid effects in human adipocytes. To validate our method with other genes we also knocked out peroxisome proliferator-activated receptor gamma (PPARG), a master regulator of adipogenesis.

## Results

### Ribonucleoprotein (RNP) complex delivered by electroporation effectively knocked out *FKBP5* and *PPARG* in human primary preadipocytes

As a proof-of-concept, the *FKBP5* gene was deleted to establish CRISPR/Cas9 gene editing in isolated human primary preadipocytes. To check whether our method is suitable for editing other genes, we also knocked out a well-established adipocyte-specific gene, *PPARG*, a master regulator of adipogenesis.

#### Assessment of *FKBP5* gene knockout

**Figure 1:**
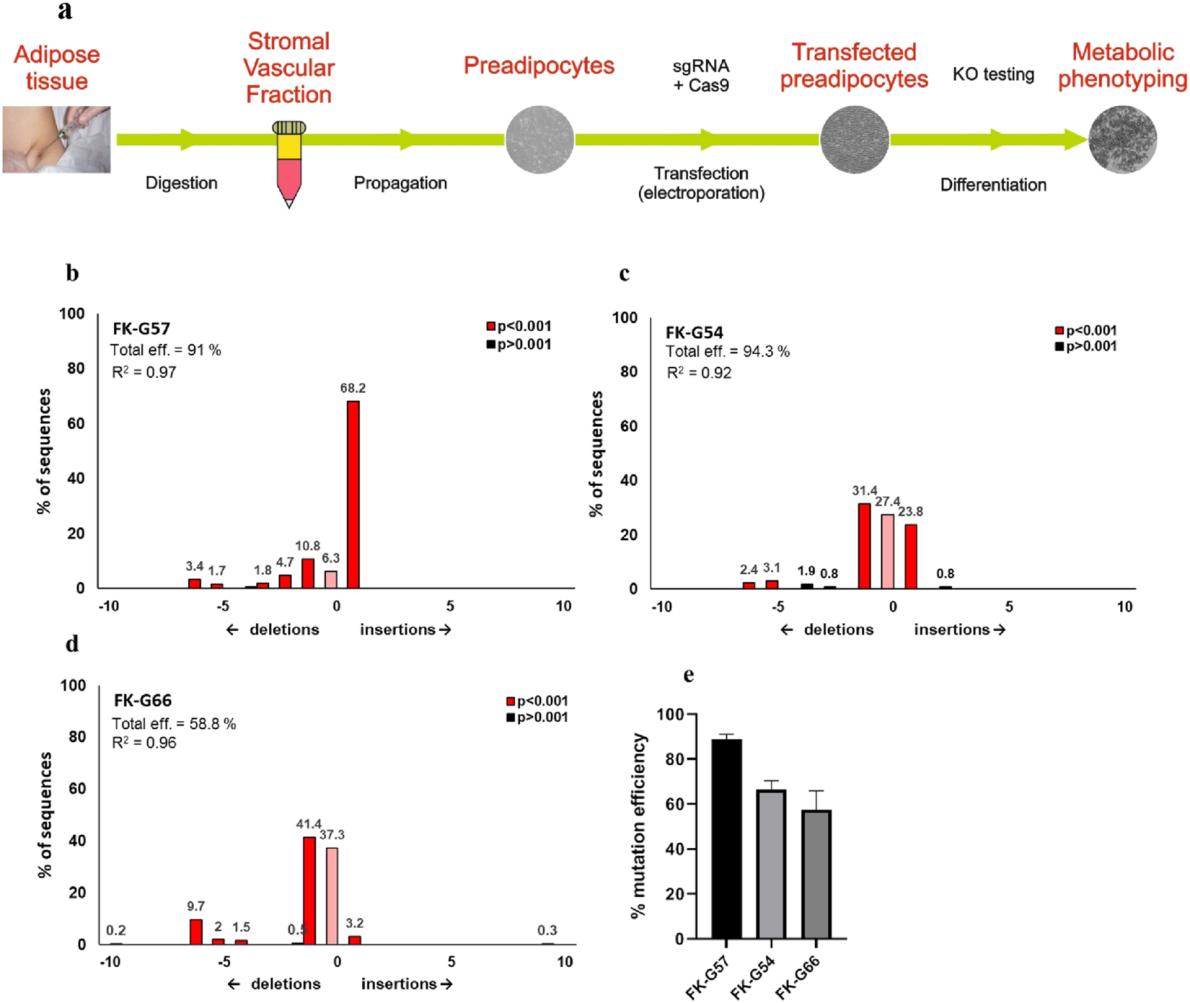
Assessment of mutation efficiency at the DNA level. **(a)** Schematic representation of the experimental setup of the whole process from collecting human adipose tissue biopsy until an assessment of knockout efficacy. (**b–e)** Quantification of Sanger sequencing chromatograms by TIDE (Tracking of Indels by Decomposition) of representative transfection experiments of SVF cells transfected with the (**b**) FK-G57 guide, (**c**) FK-G54 guide and (**d**) FK-G66 guide and sequenced in both directions. Similar mutation results were obtained independently of the DNA strand that was sequenced. The size distribution of the insertions (plus) and deletions (minus) is shown on the x-axis and the percentage contribution of each indel to the total efficiency is shown on the y-axis. R^2^ is the correlation coefficient calculated to assess the goodness of fit, and p is the estimated probability of each mutation event. (**e**) Average total mutation efficiency of 2 to 4 independent transfection experiments. Data are shown as mean ± SEM.

**Figure 2:**
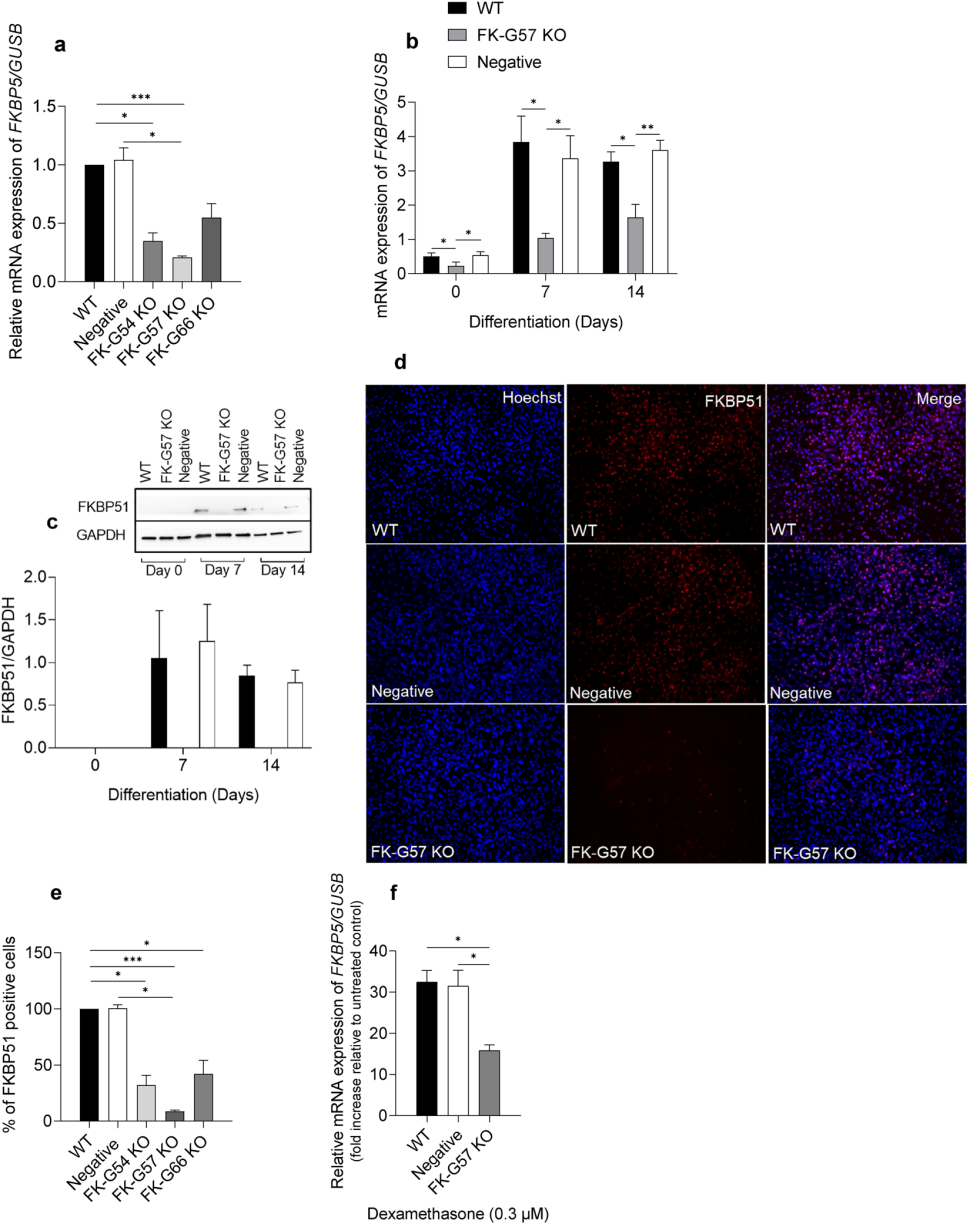
FKBP5 gene deletion and assessment of knockout efficacy. **(a)** *FKBP5* mRNA levels in wild type, negative control, FK-G54 KO, FK-G57 KO, and FK-G66 KO cultures 48 hours after transfection. *GUSB* was used as a housekeeping gene. The qPCR data were quantified using 2^-dCt^ and shown as relative to wild type (n = 3 to 5) **(b)** *FKBP5* gene expression in wild type, FK-G57 KO, and negative control cultures on days 0, 7 and 14 of differentiation. (**c**) FKBP51 protein levels in wild type and FK-G57 KO and negative control cultures on days 0, 7, and 14 of differentiation. A representative blot, along with the quantification graph, is shown. GAPDH was used as an endogenous control (n = 3). The blots are the cropped images from different parts of the same gel. The uncropped gel images are provided in the supplementary file. (**d)** Representative immunostaining images comparing the protein levels of FKBP51 in differentiated cells from wild type, negative control, and FK-G57 KO cultures. The nuclei are stained with Hoechst 33342 (blue) and FKBP51 stained with Alexa Fluor® 594 (red). (**e)** Quantification graph showing the percentage of cells positive for the FKBP51 protein in differentiated adipocytes (n = 3). (**f**) Effect of dexamethasone on the *FKBP5* gene expression between wild type, negative control, and FK-G57 KO cultures. Data are shown as mean ± SEM. *p < 0.05, **p < 0.01, ***p < 0.001. WT wild type, KO knockout.

As described in Methods, sgRNA against the *FKBP5* gene and Cas9 protein delivered by electroporation successfully knocked out *FKBP5* in isolated human primary preadipocytes. Among three different sgRNAs targeting the *FKBP5* gene, maximum knockout efficiency was achieved with FK-G57 sgRNA followed by FK-G54 and FK-G66 (Figs. 1 and 2). This was first confirmed at the DNA level (Fig. 1b–e) and also by measuring the mRNA levels of *FKBP5* in preadipocytes (Fig. 2a) after 48 hours of transfection. The knockout efficiency assessed by Sanger sequencing revealed that the FK-G57 sgRNA achieved the highest mutation efficiency compared to FK-G54 and FK-G66 (91% vs 64% and 59% for FK-G57 vs FK-G54 and FK-G66, respectively, Fig. 1b–e). Compared to wild type cultures, the mRNA levels of *FKBP5* in FK-G54, FK-G57, and FK-G66 knockout cultures were decreased by 65% (n = 3, p < 0.05), 80% (n = 5, p < 0.01), and 50% (n = 3, p = 0.15), respectively (Fig. 2a). In addition, compared to wild type cultures, the expression of *FKBP5* remained significantly lower on days 0, 7, and 14 of differentiation in FK-G57 knockout cultures (n = 5, Fig. 2b). Western blot data showed that FKBP51 protein levels were undetectable in FK-G57 knockout cultures compared to wild type on days 0, 7, and 14 of differentiation (n = 3, Fig. 2c). In agreement with Western blot, immunocytochemistry data further confirmed the loss of FKBP51 in FK-G57 knockout cultures compared to wild type (n = 3, Fig. 2d,e). Dexamethasone treatment of differentiated adipocytes from wild type cultures increased the *FKBP5* mRNA levels by 30-fold (n = 3, p < 0.05) compared to untreated controls (data not shown), whereas it was reduced by 50% in FK-G57 knockout cultures compared to dexamethasone-treated wild type cells (n = 3, p < 0.05, Fig. 2f). Given the highest knockout efficiency with FK-G57 sgRNA all other experiments were performed using this sgRNA.

#### Assessment of *PPARG* gene knockout

**Figure 3:**
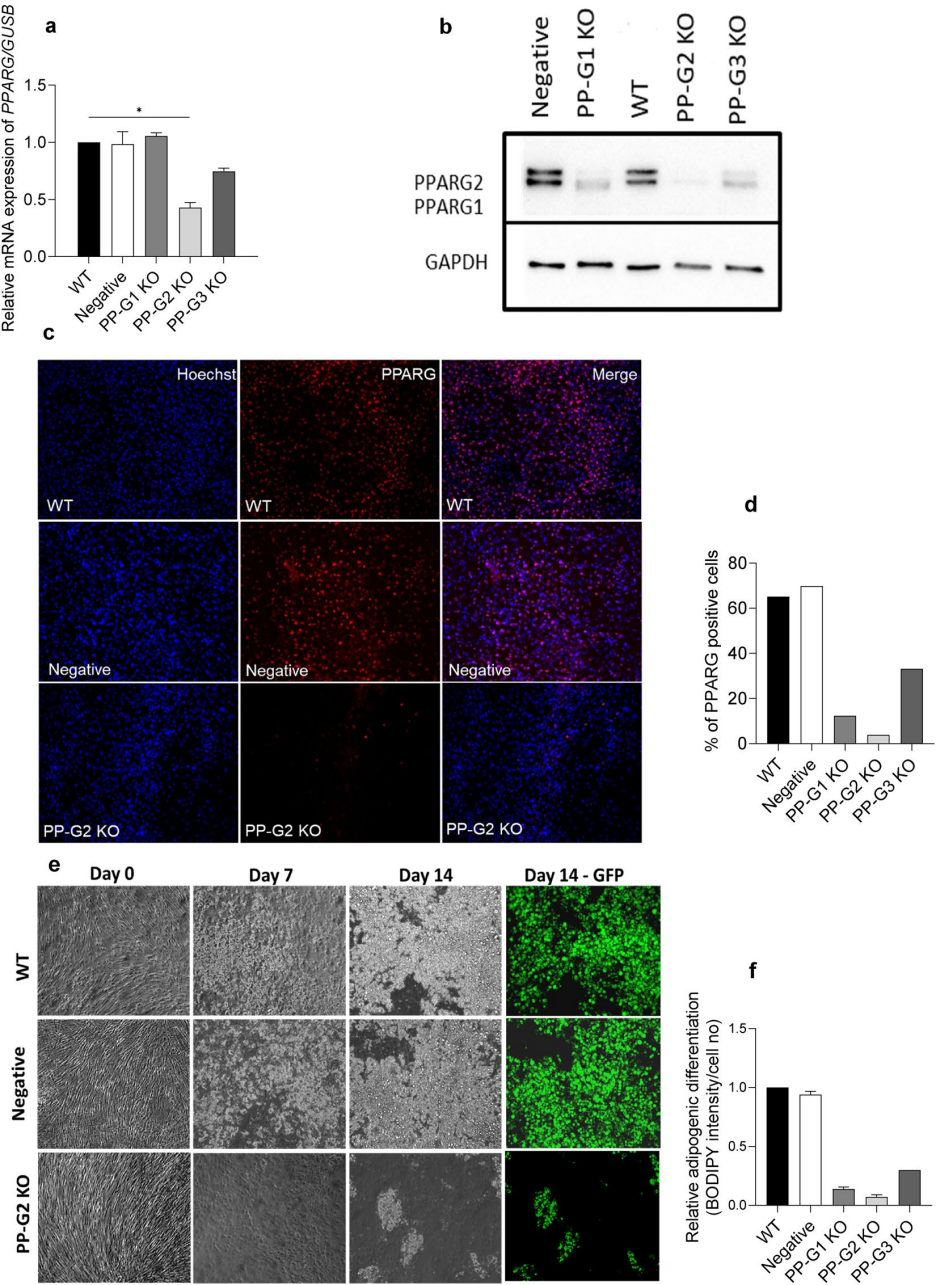
Deletion of PPARG, assessment of mutation and adipogenesis. **(a)** *PPARG* mRNA levels in wild type, negative control, PP-G1 KO, PP-G2 KO, and PP-G3 KO cultures 48 hours post-transfection. *GUSB* was used as a housekeeping gene. The data were quantified using 2^−dCt^ and shown as relative to wild type (n = 2) (**b**) A representative western blot image showing the protein levels of PPARG on day 14 of differentiation. GAPDH was used as an endogenous control. The blots are the cropped images from different parts of the same gel. The uncropped gel images are provided in the supplementary file. (**c**) Immunocytochemistry showing the loss of PPARG protein in differentiating adipocytes on day 7. The nuclei is stained with Hoechst 33342 (blue) and PPARG protein is stained with Alexa Fluor® 594 (red). (**d)** Quantification graph showing the percentage of cells positive for PPARG protein when counting >800 cells in representative cultures from one transfection experiment. (**e)** A representative bright-field image from wild type, negative control, and PP-G2 KO cultures depicting the progress of differentiation. For visualization purposes, the neutral lipids on day 14 of differentiation were stained with BODIPY and imaged under the GFP channel (green). (**f)** A graph showing the quantification of lipid accumulation on day 14 of differentiation (n = 1 to 3). WT wild type, KO knockout.

**Figure 4:**
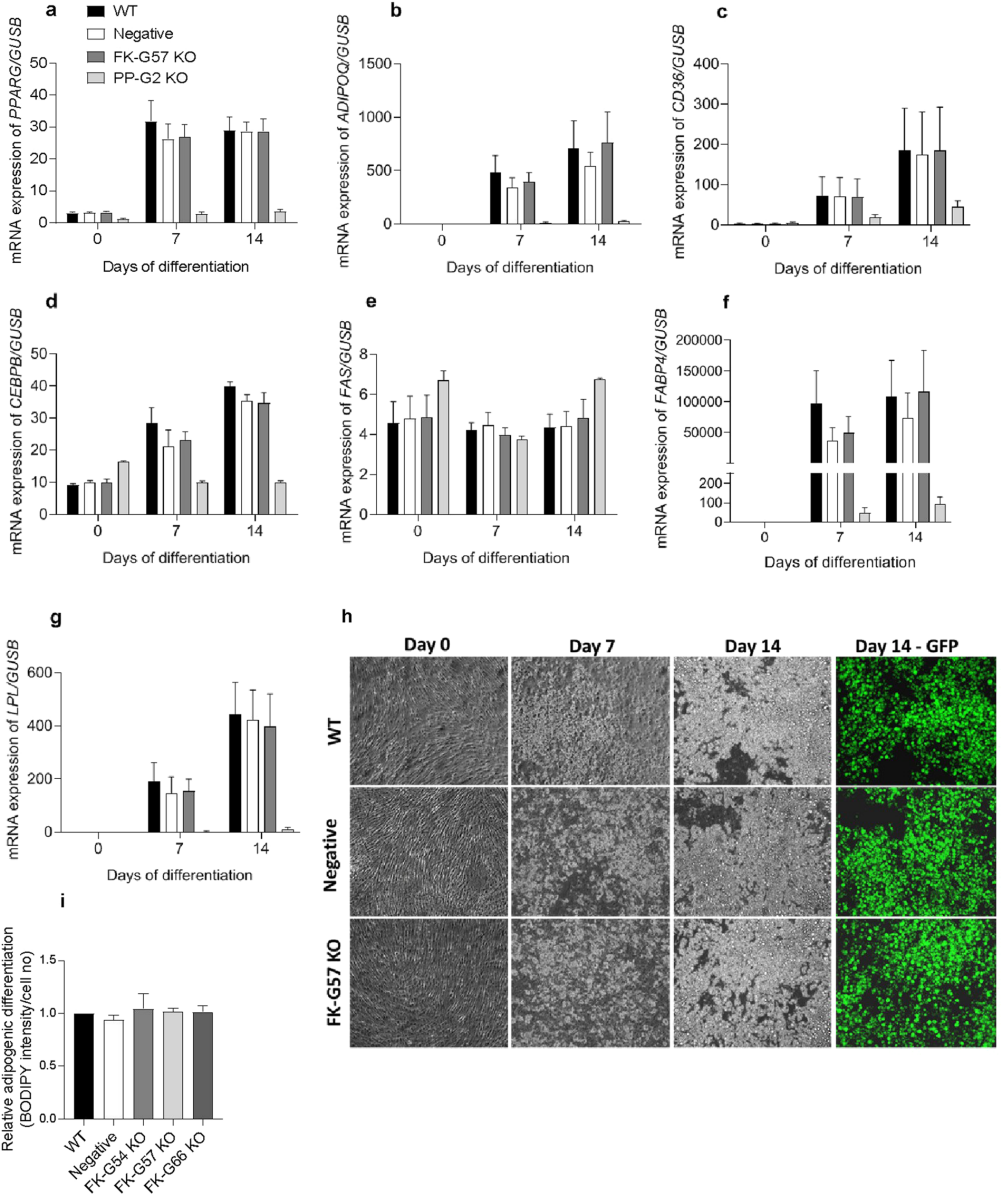
The loss of FKBP51 did not affect adipogenesis. Preadipocytes from wild type, FKBP51 (FK-G57) and PPARG (PP-G2) knockout cultures were differentiated for 14 days. (**a–g**) Differentiation was assessed by measuring the expression of adipogenic markers on days 0, 7, and 14 (n = 5). As a positive control for differentiation, the expression of adipogenic markers was also investigated from the PP-G2 knockout cultures (n = 2). (**h**) The degree of differentiation was evaluated by quantifying the amount of lipid accumulation on day 14 of differentiation. A representative bright-field image from wild type, negative control, and FK-G57 KO cultures depicting the progress of differentiation. For visualization purposes, the neutral lipids on day 14 of differentiation were stained with BODIPY and imaged under the GFP channel (green). (**i)** Quantification of lipid accumulation on day 14 of differentiation between wild type, negative control, and different FKBP51 knockout cultures. WT wild type, KO knockout.

As an experimental (method validation) control, we also knocked out the *PPARG* gene using three different sgRNAs (n = 2). Maximum efficiency was achieved with the PP-G2 sgRNA (Fig. 3a). Post 48 hours of transfection, the mRNA levels of *PPARG* in PP-G2 and PP-G3 knockout cultures were reduced by 60% (p < 0.05) and 25% (p = NS), respectively compared to wild type cultures (Fig. 3a). Cells from the PP-G1 knockout cultures did not show any reduction in the *PPARG* gene expression, which could be due to the transcription of CRISPR/Cas9 edited DNA into modified mRNA and its detection by the PCR primers used. Nevertheless, the protein level is generally a better measure for assessing the true level of knockdown from a functional perspective. The preliminary data shows that compared to wild type cultures, the protein expression of PPARG1 and PPARG2 isoforms on day 14 of differentiation were reduced in all three knockout cultures with maximum reduction achieved in PP-G2 knockout culture (reduction in both isoforms by at least 90%) (Fig. 3b). Immunostaining data also confirms the loss of PPARG in PP-G2 knockout cultures compared to wild type (Fig. 3c,d). As expected, the loss of PPARG inhibited adipogenesis, assessed by quantifying the lipid accumulation (Fig. 3e,f) and expression of adipogenic markers (Fig. 4a–g). Of note, the cells from both wild type and knockout cultures reached confluence on the same day. Besides, the number of cells determined on the 14^th^ day of differentiation was similar between cultures, suggesting that the observed differences in the differentiation were due to the loss of PPARG and not due to the reduced proliferation capacity of edited cells (Supplementary material, Fig. 2).

Preadipocytes transfected with a non-coding sgRNA (negative control) did not show any change in the expression of either FKBP51 or PPARG (Figs. 1–3).

### Loss of FKBP51 did not affect adipogenesis

There was no difference in the differentiation capacity of preadipocytes between wild type and FKBP51 knockout cultures (Fig. 4). The rate of differentiation assessed by measuring the expression of adipogenic markers such as *PPARG, ADIPOQ, CD36, CEBPB, FAS, FABP4*, and *LPL* at different time points during differentiation remained similar between wild type and FK-G57 knockout cultures (n = 5, Fig. 4a–g). Likewise, the degree of differentiation measured by quantifying lipid accumulation on day 14 of differentiation did not change between wild type and FK-G57 knockout cultures (n = 3 to 5, Fig. 4h,i).

### FKBP51 may mediate dexamethasone’s inhibitory effect on adipocyte glucose uptake capacity

**Figure 5:**
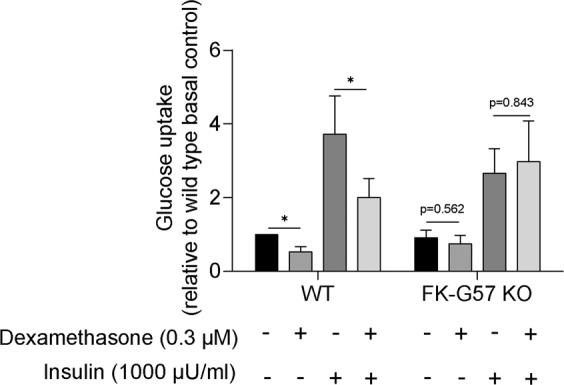
Effect of dexamethasone on glucose uptake in differentiated adipocytes from wild type and FKBP51 knockout (FK-G57) cultures. Differentiated adipocytes were treated with or without dexamethasone (0.3 µM) for 24 hours, and basal and insulin-stimulated (1000 µU/ml) glucose uptake was measured (n = 6). The basal glucose uptake in wild type cultures was 9.0 ± 5.0 femtoliter/mg/s and in FK-G57 knockout cultures 5.0 ± 1.0 femtoliter/mg/s. Data are shown as mean ± SEM. *p < 0.05.

At the end of differentiation, matured adipocytes were maintained in media without any glucocorticoids for 48 hours, followed by treatment with or without dexamethasone (0.3 µM), and glucose uptake was performed (n = 6, Fig. 5). As expected, in wild type cultures, dexamethasone treatment significantly reduced basal and insulin-stimulated (1000 µU/ml) glucose uptake by ~50% compared to untreated cultures (p < 0.05). In contrast to wild type cultures, the inhibitory effect of dexamethasone was abrogated in FK-G57 knockout cultures.

### FKBP51 may regulate transcriptional activity of glucocorticoids in adipocytes

**Figure 6:**
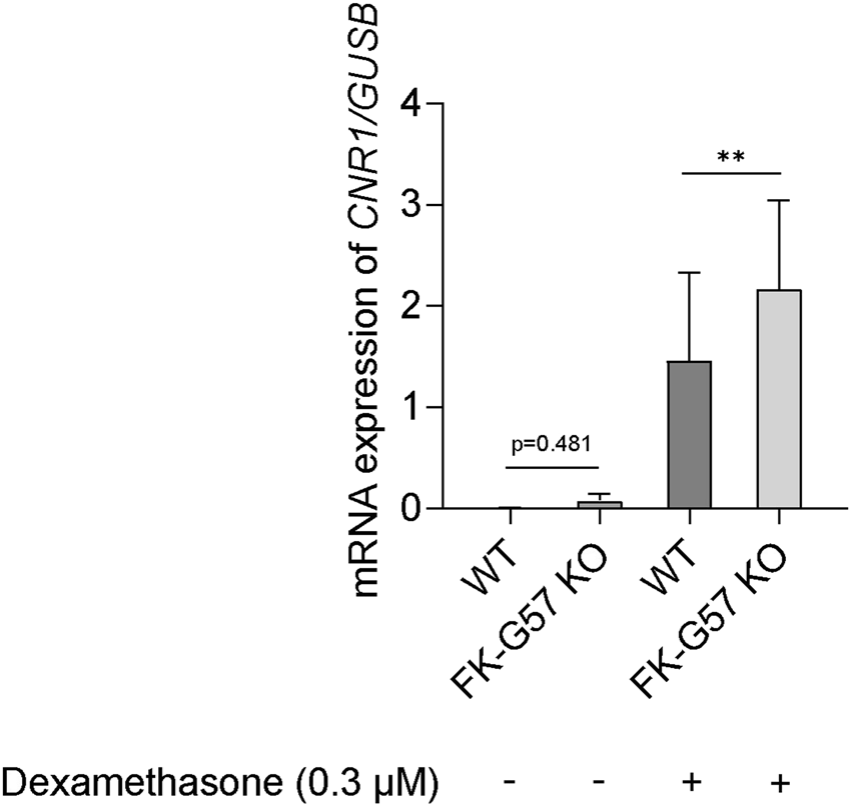
Dexamethasone increased *CRN1* gene expression in FKBP51 knockout (FK-G57) adipocytes. Differentiated adipocytes were treated with or without dexamethasone (0.3 µM) for 24 hours. RNA was extracted, and the expression of the *CNR1* gene was assessed using qPCR (n = 6). *GUSB* was used as an endogenous control. Data are shown as mean ± SEM. **p < 0.01.

Differentiated adipocytes from wild type and FKBP51 knockout (FK-G57) cultures were maintained in medium without any glucocorticoids for 48 hours. Thereafter, cells were treated with or without dexamethasone (0.3 µM) for 24 hours, and mRNA expression of *CNR1*, a glucocorticoid-regulated gene, was measured. In untreated samples, *CNR1* gene expression was detected in 2 out of 6 samples, and it was non-significantly higher in FK-G57 knockout cultures than wild type. Therefore, we mainly compared the *CNR1* expression in response to dexamethasone, which was about 50% higher in FK-G57 knockout cultures than wild type (p < 0.01, n = 6, Fig. 6). Dexamethasone itself markedly stimulated CNR 1 expression in WT (p < 0.05) as well as FK-G57 (p < 0.01) cells. In addition, we assessed expression of other genes that are known to be regulated by glucocorticoids in adipose tissue^6^ such as *PDK4, LPL, LEP, TIMP4* and *IL6*, but we did not find any difference in their expression in response to dexamethasone between wild type and FK-G57 knockout cultures (data not shown).

## Discussion

Here, we describe a simple method to effectively knockout genes in human primary preadipocytes using CRISPR/Cas9 technology. As a proof-of-concept, we knocked out the *FKBP5* gene and studied its effects on human adipogenesis, as well as assessed whether it mediates glucocorticoid action in human adipocytes. We show that chemically modified guide RNA and Cas9 protein in the form of an RNP complex delivered into the cells using electroporation, under the current experimental conditions can effectively knockout genes in human primary preadipocytes. Moreover, this was achieved with a transfection and knockout efficiency high enough to eliminate the need for selection of edited cells or clonal isolation after transfection. This is especially important while handling human primary preadipocytes as they lose their differentiation potency after undergoing several rounds of cell division, unlike other adipose cell models such as 3T3-L1 or SGBS cells that retain the capacity for a much higher number of passages^10,11^. A low transfection/transduction rate would result in a very low yield of cells after selection, which may then have to be proliferated longer to achieve a reasonable number of cells, depending on the size of the subsequent experimental set-up. This could lead to a discrepancy in the passage number between wild type control cells and the transfected cells, which might result in false-positives unless proper transfection controls are included. For instance, a loss of adipogenic potency of the transfected preadipocytes may not be due to the actual study intervention, but could instead be due to the transfected or transduced cells having divided more times compared to wild type cells, which can be misleading. The ideal scenario would be to have a large number of cells as starting material and an optimal method that can result in a high transfection/transduction efficiency, where there would not be any differences in cell number between control cultures and the transfected cultures.

To the best of our knowledge, only one study has reported using CRISPR/Cas9 technology to introduce point mutation in a gene in primary human adipose cells^4^. However, in that study, authors have used an expression vector to deliver CRISPR components into cells. As discussed in the literature^12^, there are potential issues associated with the use of plasmid DNA including the suitability of cells for transfection or viral transduction, a requirement of an appropriate promoter for both Cas9 and gRNA expression, as well as the incorporation of plasmid DNA into the genome and the possibility of high off-target effects due to prolonged Cas9 expression. Since Cas9 has first to be transcribed and translated, it delays the procedure. One alternative option, to circumvent many of these complications, is to deliver an RNP complex, comprising the Cas9 protein and a single guide RNA (sgRNA), directly into cells^12^. There are several advantages of using this system over plasmid DNA or viral-based delivery systems. Due to the RNP complex being rapidly degraded by the cell post-delivery, the effects are transient and the Cas9 enzyme activity would be restricted and cannot continue to cut the DNA indefinitely, thereby potentially decreasing off-target effects. This method can be used not just for straightforward knockout experiments, but can also be adapted for base editing^13^, as well as for the introduction of selection markers^14^. However, in our study, we chose not to include selection markers since we were able to achieve at least 90% knockout efficiency without enriching for edited cells. This is an advantage for primary preadipocytes as it reduces the time from transfection to the initiation of adipogenesis and hence the cells retain a high differentiation capacity.

For initial experiments, all three guides for each gene were included not only for knockout efficiency estimation but also for the key phenotyping assays to verify consistent effects and rule out potential off-target effects.

To avoid potential problems associated with the plasmid vector, we opted for delivering CRISPR components into cells as an RNP complex. To optimize the electroporation protocol, we transfected preadipocytes with sgRNA targeting HPRT locus and Cas9 protein and used different combinations of electroporation voltage and number of pulses. The locus-specific genomic cleavage was detected as a simple readout of percent insertion or deletion (indel). In 4 out of 8 combinations, we found more than 40% indel. The combination of voltage and the number of pulses that resulted in the highest indel efficiency was then used to transfect preadipocytes for the deletion of genes of interest. In the future, it would be interesting to test other combinations as well.

To test the efficiency of the current method to knockout other genes in human primary preadipocytes we also knocked out the *PPARG* gene. It also provided a positive control for modifying adipogenesis. Interestingly, we achieved over 90% inhibition of both PPARG1 and PPARG2 protein isoforms in knockout cultures compared to wild type. Moreover, as expected, the differentiation capacity of cells from knockout culture was markedly inhibited when using the best performing guide RNA. This suggests that the method achieved excellent transfection efficiency. Also, there was a clear relationship between the effect on differentiation and the level of PPARG knockout, which validates the guide RNAs used, and confirms the critical role of PPARG for adipocyte differentiation.

We observed that the *FKBP5* gene and protein expression was upregulated during adipogenesis, which is in agreement with our previous publication^15^. However, in confluent preadipocytes, the corresponding protein levels were below detection limits. A previous study has suggested that FKBP51 regulates PPARG activity via the Akt-p38 kinase pathway, which potentially affects adipogenesis^16^. It has been demonstrated that FKBP51 knockdown in 3T3-L1 cells and knockout in mouse embryonic fibroblast impairs adipogenesis^17,18^. In contrast, a study by Toneatto *et al*., showed that in 3T3-L1 cells, FKBP51 knockdown facilitates, whereas its ectopic expression blocks adipogenesis^19^. In line with Toneatto *et al*., we recently reported that *FKBP5* gene expression in subcutaneous adipose tissue from both, non-diabetic and type 2 diabetes subjects, was inversely associated with the expression of adipogenic markers such as *PPARG* and *CEBPB*^15^. However, the cross-sectional nature of that study does not take into account the time course or causality. In the present study, we show that knocking out FKBP51 in human adipose tissue-derived preadipocytes does not affect their adipogenic potential when compared to wild type cells. Moreover, in contrast to the studies mentioned above^17–19^ both the expression of adipogenic markers and lipid accumulation at the end of differentiation did not change between two cultures. These findings were also replicated in another human adipocyte cell model, the SGBS cells (data not shown). A possible explanation for the observed discrepancies between our findings and previously published results^17–19^ could be the use of different experimental models and the different methods selected for gene-editing. Nevertheless, the use of primary human adipose tissue-derived preadipocytes is a preferable method to reflect the *in vivo* situation in humans closely and our results suggest that FKBP51 may not be crucial for adipogenesis in humans. As a positive control, we also knocked out the *PPARG* gene in preadipocytes collected from the same subjects as to knockout the *FKBP5* gene. As expected, preadipocytes from the PPARG knockout cultures did not differentiate compared to wild type cultures^20^, which further strengthens our findings that FKBP51 is not involved in human adipogenesis.

FKBP51 is a co-chaperone of HSP90 and mainly functions as a negative regulator of the glucocorticoid receptor activity via an intracellular negative feedback mechanism^21^. In adipose tissue, glucocorticoids have been shown to cause insulin resistance, for instance, by reducing glucose uptake capacity of adipocytes^6^. To better understand whether FKBP51 is a factor mediating the inhibitory effect of glucocorticoids on glucose uptake, differentiated adipocytes from wild type and FKBP51 knockout cultures were treated with dexamethasone, and their glucose uptake capacity was assessed. As expected, dexamethasone exposure significantly decreased basal and insulin-stimulated glucose uptake in adipocytes from wild type cultures. However, the inhibitory effect of dexamethasone was abrogated in FKBP51 knockout cultures, suggesting that FKBP51 may mediate glucocorticoid action to inhibit glucose uptake in adipocytes. These results are in agreement with our recent study in which an FKBP51 specific inhibitor partially prevented the inhibitory effect of dexamethasone on glucose uptake in human adipocytes^15^. A recent study in mice showed that genetic or pharmacological blockade of FKBP51 inhibited glucose uptake in primary myotubes, suggesting its role in glucose uptake in muscle cells^22^. However, in our study deletion of FKBP51 in adipocytes by itself did not affect glucose uptake. Taken together, our data suggest that in human adipocytes, FKBP51 alone may not interfere with glucose uptake, but it may be involved in glucocorticoid action.

To understand the role of FKBP51 to modify glucocorticoid action in adipocytes, we asked whether the loss of FKBP51 alters the transcriptional activity of the glucocorticoid receptor and thereby its action. We addressed this question by measuring the expression levels of several previously reported glucocorticoid target genes in differentiated adipocytes^6,23^. However, we observed that only *CNR1* gene expression was differentially expressed following dexamethasone exposure in wild type vs FKBP51 knockout cultures, respectively. This implies that FKBP51 does not modify all transcriptional actions of glucocorticoids similarly, but rather in a selective manner.

In untreated control samples, *CNR1* gene expression tended to be higher in FK-G57 knockout cultures than wild type. However, in FKBP51 knockout cultures, dexamethasone augmented expression of the *CNR1* gene compared to wild type cultures. This suggests that FKBP51 may have a regulatory role in glucocorticoid transcriptional action. At the molecular level, FKBP51 has been shown to interact with glucocorticoid receptors and their downstream signaling^24^. The FKBP51 glucocorticoid receptor complex has a low affinity towards glucocorticoids and thus reduced nuclear translocation and transcriptional action. Hence, the loss of FKBP51 may be expected to increase glucocorticoid action. This notion is supported by the observed increase in *CNR1* expression by dexamethasone in FKBP51 knockout cultures compared to wild type. In line with this, the effect of dexamethasone to inhibit glucose uptake would also be expected to be enhanced in FKBP51 knockout cultures. However, FKBP51 deletion instead prevented dexamethasone’s inhibitory effects on glucose uptake, possibly via non-genomic pathways^25^. As mentioned above, a recent study showed that the stimulating effects on glucose uptake in FKBP51 knockout myotubes involve activation of AKT2-AS160 signaling^22^. Elucidating whether the same mechanisms also occur in human adipocytes would be of interest.

There are some limitations to take into account. Firstly, we included only female subjects; however, this was mainly to avoid any confounding effects due to previously shown sex-specific effects of glucocorticoids on adipose tissue^26,27^. Therefore, future studies should also include male subjects to investigate the effects of dexamethasone observed in the current study. The interaction between the glucocorticoid receptor and FKBP51 is not thoroughly explored. However, this study primarily aimed to establish the CRISPR technique and establish basic phenotypes of the FKBP51 and PPARG knockout in human adipose cells. We plan further work to explore the interplay between FKBP51 and glucocorticoids in more detail. This will address genomic as well as non-genomic actions of glucocorticoids^25^ and the latter may very well be critical for the interactions with glucose transport. The insertion of random indels might result in off-targets or introduce undesirable epigenetic modifications within the gene locus, which can lead to false positives. Ideally, whole genome sequencing would be needed to identify such changes in the genome, and this would be very tedious and costly. Thus, to mitigate this issue we confirmed the key phenotypes using three different sgRNAs against the target gene, and the results were comparable. This supports that the phenotypes we observed were due to a reduction in the level of our proteins of interest rather than to off-target effects. Lastly, it would also be important to test the validity of the method presented here by knocking out genes other than those we studied in human primary preadipocytes.

In summary, we have established a method for gene knockout studies in human primary preadipocytes using CRISPR/Cas9. As a proof-of-concept, we knocked out *FKBP5* and *PPARG* genes in human primary preadipocytes, which resulted in at least 90% deletion of their corresponding proteins, FKBP51 and PPARG, respectively. Importantly, this was achieved without selecting for the transfected cells. Furthermore, we show that, contrary to results in animal cell models, the loss of FKBP51 did not affect the differentiation of human primary preadipocytes. This was further supported by adipogenesis being inhibited, as expected, in PPARG knocked out cells, confirming the validity of the approach. Lastly, we studied the interaction between glucocorticoid action and FKBP51 in differentiated knockout and wild type adipocytes and showed that FKBP51 can be a modulator of glucocorticoid effects in human adipocytes. However, further research is needed to fully clarify their interactions, which have potential implications for the development of insulin resistance and type 2 diabetes.

In conclusion, our method is simple, easy to implement, and enables the use of native human primary preadipocytes instead of modified cell lines and animal adipose cells. This will be important for further investigation of key genes and their products in adipose development and metabolism and understanding of adipose factors contributing to dysregulated energy metabolism in obesity and type 2 diabetes.

## Methods

All methods were carried out in accordance with relevant guidelines and regulations.

### Subjects

In total, eight women participated in the study (age: 20–71 years; body mass index: 24.2–43.1 kg/m^2^). The standard criteria were followed for the inclusion of subjects as described previously^28^. Briefly, subjects arrived in the morning after an overnight fast at the diabetes outpatient clinic at Uppsala University Hospital, Uppsala, Sweden. Anthropometric measurements were performed, and fasting blood samples were taken to determine levels of plasma glucose and lipids and serum insulin and C-peptide at the Department of Clinical Chemistry, Uppsala University Hospital. Abdominal subcutaneous adipose tissue was obtained by needle aspiration after administration of local anesthetic lidocaine (Xylocain; AstraZeneca, Sweden) in the lower two quadrants of the abdomen. Subjects with diabetes and other endocrine disorders, cancer or other major illnesses, as well as ongoing medication with beta-adrenergic blockers, systemic glucocorticoids or immune-modulating therapies were excluded from the study. The anthropometric and biochemical characteristics of the subjects are presented in Table 1. The Regional Ethics Review Board in Uppsala approved the studies (Dnr 2013/330 and Dnr 2013-183/494), and all participants gave their written informed consent.

**Table 1:** Anthropometric and clinical characteristics of subjects included in the study.

Subject characteristics (n = 8)	Mean (SD)
Age (years)	34 (±16)
Gender (m:f)	0:8
Weight (kg)	85.4 (±19.5)
BMI (kg/m^2^)	30.5 (±6.3)
Waist-hip ratio	0.8 (±0.1)
Body fat (%)	37.8 (±8.5)
Fat free mass (kg)	51.6 (±4.8)
Plasma glucose (mmol/L)	5.5 (±0.5)
HbA1c (mmol/mol)	33.3 (±2.5)
Serum insulin (µU/ml)	11.3 (±4.0)
Serum C-peptide (nmol/L)	0.8 (±0.3)
Plasma cholesterol (mmol/L)	4.4 (±1.4)
Plasma HDL-cholesterol (mmol/L)	1.4 (±0.2)
Plasma LDL-cholesterol (mmol/L)	3.0 (±1.0)
Plasma triglycerides (mmol/L)	0.9 (±0.3)

BMI body mass index, WHR waist-hip ratio, HbA1c glycated hemoglobin,

HDL high-density lipoprotein, LDL low-density lipoprotein.

### CRISPR/Cas9 mediated gene knockout in human adipose tissue-derived preadipocytes

The protocol for CRISPR/Cas9 mediated deletion of *FKBP5* and *PPARG* genes was optimized using preadipocytes derived from the stromal vascular fraction (SVF). Here, we describe the steps from obtaining human adipose tissue biopsies until an assessment of knockout efficiency. A brief overview of the method is shown in Fig. 1a.

#### Human adipose tissue: isolation and expansion of preadipocytes

Adipose tissue (4 to 6 grams) was collected in sterile conditions in Hank’s medium (Medium 199, Gibco, Life Technologies, Paisley, UK) supplemented with 5.6 mM glucose, 4% bovine serum albumin (BSA, Sigma, MO, USA), 150 nM adenosine (Sigma, MO, USA), pH 7.4 and maintained at 37 °C. Tissue was digested with collagenase (1.2 mg/ml, from *Clostridium histolyticum*, Roche, Manheim, Germany) in Hank’s medium (5.6 mM glucose, 4% BSA, 150 nM adenosine) for 60 minutes in a shaking water bath at 37 °C and 105 RPM. The cell suspension was collected into a falcon tube after filtering it through a nylon mesh and let it rest for 5 minutes to allow adipocytes to float. The SVF containing preadipocytes was separated from mature adipocytes by needle aspiration and transferred into another falcon tube. The SVF was centrifuged at 1200 RPM for 3 minutes. The pellet was resuspended in the red cell lysis buffer (0.154 M NH_4_Cl, 10 mM KHCO_3,_ and 0.1 mM EDTA, all from Sigma) and centrifuged again at 1200 RPM for 3 minutes. The pellet was then cultured in 75 cm^2^ Nunc™ EasYFlask™ Cell Culture Flasks (Thermo Fisher) with preadipocyte medium (Dulbecco’s modified Eagle’s medium (DMEM)/Ham’s F12, Gibco) containing 10% foetal bovine serum (FBS, Thermo Fisher), 100 units/ml penicillin and 100 g/ml streptomycin (PEST, Life Technology), 0.04 mg/ml gentamycin (Gibco), and 4.125 ng/ml basic fibroblast growth factor (bFGF) (Sigma) at 37 °C. The next day, the medium was replaced and isolated preadipocytes were grown until 70–80% confluence (Passage 0). From this, preadipocytes were trypsinized and propagated into two 175 cm^2^ Nunc™ EasYFlask™ Cell Culture Flasks (Thermo Fisher) (Until passage 2–3). Preadipocytes from passage 2–3 were used for gene deletion.

#### Design and selection of a single guide RNA

Single guide RNAs (sgRNAs) targeting the human *FKBP5* and *PPARG* genes were designed either using the Broad institute RNA guide design web tool (https://portals.broadinstitute.org/gpp/public/analysis-tools/sgrna-design) or obtained from Integrated DNA Technologies (IDT) and Thermo Fisher as pre-existing designs. All guide RNA designs were also evaluated by standard guide RNA design considerations before chosen for experiments^29^. The efficiency of three sgRNAs was assessed (see results under assessment of knockout efficiency section) for each gene after which the best performing guide was chosen for subsequent experiments. The sgRNA sequences for *FKBP5* (we refer to them as FK-G54, FK-G57, and FK-G66) were: 5′-GAAGACCACGACATTCCAATtgg-3′, 5′-ccaATTGGAATTGACAAAGCTCT -3′ and 5′ATCCGGAGAACCAAACGGAAagg-3′; respectively and for *PPARG* (we refer to them as PP-G1, PP-G2, and PP-G3) were: 5′- CAACTTTGGGATCAGCTCCGtgg-3′, 5′-ACGACATTCAATTGCCATGAggg-3′ and 5′-GATGGGGTTCTCATATCCGAggg-3′, respectively (protospacer adjacent motif (PAM) sequences shown in lower case letters). A non-targeting TrueGuide^TM^ sgRNA (Thermo Fisher) was included as a negative control.

#### Optimization of electroporation parameters

The electroporation parameters were optimized by transfecting adipose tissue-derived preadipocytes with sgRNA targeting the human hypoxanthine phosphoribosyltransferase (HPRT) (Thermo Fisher, Cat no: A35524) locus and Cas9 protein using a combination of different voltage and number of pulses (Supplementary Table 1). *HPRT* is a housekeeping gene and a commonly used control. The genome-editing efficiency was tested by analyzing the locus-specific cleavage of genomic DNA using GeneArt® Genomic Cleavage Detection Kit (Thermo Fisher). The details are given in the supplementary text (Supplementary Fig. 1). The optimal voltage, pulse width, and pulse number were 1750 Volts, 20 milliseconds, and 1, respectively to knock out *FKBP5* and *PPARG* genes.

#### Transfection of preadipocytes with sgRNAs targeting FKBP5 or PPARG genes and Cas9

The cells were electroporated with a Neon® Transfection system and The Neon® Transfection System 10 µL Kit (Thermo Fisher) as per the manufacturer’s guidelines with slight modification. Briefly, to form a RNP complex, chemically modified sgRNA (9.3 pmol/reaction) and TrueCut^TM^ Cas9 protein v2 (6 pmol/reaction) (both from Thermo Fisher) were mixed in buffer R and incubated for 15 minutes at room temperature. The preadipocytes (at passage 2–3) dissolved in buffer R were then mixed with the RNP complex. In total, 60 000 cells were transfected in a single electroporation reaction, and a total of about 8 reactions were performed for each guide. Following each electroporation reaction, cells were transferred into a 75 cm^2^ cell culture flask containing DMEM/Ham’s F12 supplemented with 10% FBS, but without any antibiotics, and incubated for 48 hours at 37 °C. Thereafter, the medium was changed to DMEM/Ham’s F12 supplemented with 10% FBS, 1% PEST, 0.04 mg/ml gentamycin, and 4.125 ng/ml bFGF and cells were maintained until they became 70–80% confluent. From this, some cells were collected to test knockout efficiency, and the rest were further passaged into two 175 cm^2^ flasks and maintained as described above until 70–80% confluence (passage 3–4). Wild type cells were also exposed to the electroporation treatment in buffer R, but without an RNP complex. The wild type and transfected cells from passage 4–5 were seeded for differentiation.

Knockout efficiency was tested in preadipocytes collected 48 hours post-transfection (passage 3–4), as well as at the end of differentiation at mRNA and protein levels (passage 4–5). The methods used are described below. Preadipocytes from all subjects were used for the deletion of the *FKBP5* gene, while preadipocytes from two subjects were used for the deletion of the *PPARG* gene. For functional assessment, the number of subjects used is mentioned in the results section.

#### Assessment of genomic mutation efficiency using Sanger sequencing

Genomic DNA from wild type and transfected cells were isolated using DNeasy Blood & Tissue Kit (Qiagen, Hilden, Germany). PCR amplification of the target sequence was carried out with 20 ng gDNA using Dream Taq Hot Start polymerase (Thermo Fisher) and the primers: 5′-CTGTCTCCTTGCCCTCAGTC-3′ and 5′-CGTGATGACTAACTGCAGCC-3′ for FK-G54 and FK-G66, and 5′-AAGTCTCCATTTCGTCCACATAAA-3′ and 5′-GCACTGCAGTCCCTTAGGATA-3′ for FK-G57. PCR conditions for FK-G54 and FK-G66 were: 3 min at 95 °C, followed by two touchdown cycles of 30 s at 95 °C, 20 s at 61 °C, 60 °C, 59 °C, 58 °C, 57 °C and 30 s at 72 °C and ending with 25 cycles of 30 s at 95 °C, 20 s at 56 °C and 30 s at 72 °C. PCR conditions for FK-G57: 3 min at 95 °C, followed by two touchdown cycles of 30 s at 95 °C, 20 s at 65 °C, 64 °C, 63 °C, 62 °C, 61 °C and 30 s at 72 °C and ending with 25 cycles of 30 s at 95 °C, 20 s at 60 °C and 30 s at 72 °C. PCR products were run on a 1.5% agarose gel and gel purified using the GeneJet Gel Extraction kit (Thermo Fisher). Amplicons were between 350–700 base pairs in length. Aliquots of 5 ng/µl DNA in 15 µl reactions were Sanger sequenced in both directions using the amplification primers. Independent transductions were assessed for the efficiency of genome editing using the Tracking of Indels by Decomposition (TIDE). The chromatograms were analyzed using the online tool at https://tide.nki.nl/.

#### Adipogenesis

Adipogenesis was carried out as previously described^28^. Preadipocytes at 15000 cells/cm^2^ were seeded (passage 4–5) into 12 well plates (Nunclone, Thermo Fisher) using preadipocyte media. After cells reached 100% confluence, differentiation was induced by adding a differentiation cocktail (DMEM/Ham’s F12, 1% PEST, 100 nM (16666 µU/ml) human insulin (Actrapid, Novo Nordisk, Bagsvaerd, Denmark), 17 µM pantothenate (Sigma), 33 µM biotin (Sigma), 1 µM cortisol (Sigma), 1 µM rosiglitazone (Sigma), 250 µM 3-isobutyl-1-methylxanthine (IBMX, Sigma), 10 µg/ml transferrin human (Sigma), 2 nM 3, 3, 5-triiodo L-thyronine (T3, Sigma)) for 5 days with replacement of induction cocktail after first three days. Thereafter, cells were cultured in maintenance medium (composition is similar to that of differentiation cocktail except for IBMX) until day 14. The medium was replenished every 3 days. The degree of adipocyte differentiation was assessed using fluorescent staining of lipid droplets and by measuring the expression levels of differentiation markers in cells collected on confluence (day 0), 7, and 14 days post-induction.

### Glucose uptake

Glucose uptake was performed in adipocytes differentiated *ex vivo* (n = 6). On day 14, differentiated adipocytes were maintained for 48 hours without any glucocorticoids, thereafter adipocytes were treated for 24 hours with or without 0.3 µM dexamethasone (Sigma) and glucose uptake was performed as previously described^11^. Briefly, following dexamethasone treatment, cells were washed two times with warmed PBS and then incubated for two hours in Krebs Ringer HEPES (KRH) buffer containing 0.01% BSA (Sigma), with 5 mM glucose (Sigma), 200 nM adenosine (Sigma) and pH 7.4. Thereafter, cells were washed with KRH without glucose two times and maintained in the same buffer until the next step. To assess basal and insulin-stimulated glucose uptake, cells were incubated for 30 minutes without or with insulin (1000 µU/ml) followed by incubation with D-[U-14C] glucose (0.26 mCi/L, 0.86 µM; PerkinElmer, Waltham, Massachusetts, USA) for 10 minutes. The reaction was stopped by transferring the plates onto the ice. The cells were washed three times with ice-cold PBS and lysed with 1% Triton-X 100 buffer. The radioactivity in the lysate was then measured using a liquid Scintillation Analyzer (Perkin Elmer, MA, USA). Glucose uptake was determined by the rate of transmembrane glucose transport and was normalized to total protein amount in each condition and shown as relative to basal control from wild type cultures. Each condition was run in triplicate.

### Gene expression analysis

The analysis of gene expression was carried out as described previously^28^. Total RNA was extracted from preadipocytes and days 7, and 14 post-induction of differentiation using the RNeasy lipid tissue mini kit (Qiagen, Hilden, Germany) and reverse transcribed using High Capacity cDNA Reverse Transcriptase Kit (Applied Biosystems, Foster City, CA, USA). The protocol was carried out as per manufacturer guidelines. The concentration and purity of total RNA were measured with the Nanodrop (Thermo Scientific). TaqMan gene expression assays (Thermo Fisher) were used to study knockout efficiency for *FKBP5* (Hs01561006) and *PPARG* (Hs01115513), and the expression levels of the differentiation markers *CEBPB* (Hs00942496)*, CD36* (Hs00169627)*, FAS* (Hs00163653)*, ADIPOQ* (Hs00605917), *FABP4* (Hs01086177), and *LPL* (Hs00173425) and *CNR1* (Hs01038522) in response to dexamethasone in differentiated adipocytes. The gene expression was detected using a QuantStudio 3 sequence detection system (Applied Biosystem). The data were calculated using a 2^−dCt^ method as previously described^30^. The results are plotted as relative quantification using *GUSB* as an endogenous control. All samples were run in duplicates.

### Western blot

Total proteins were extracted from preadipocytes or days 7 and 14 post-induction of differentiation using lysis buffer (25 mM Tris-HCl; 0.5 mM EGTA; 25 mM NaCl; 1% Nonidet P-40; 1 mM Na_3_VO_4_; 10 mM NaF (all from Sigma); 100 nM okadaic acid (Alexis Biochemicals, Lausen, Switzerland), 1X Complete protease inhibitor cocktail (Roche, Indianapolis, IN, USA), and pH 7.4). The sample was rocked for 30 minutes at 4 °C and centrifuged at 12 000 g for 15 min at 4 °C. The lysate was collected, and the protein concentration was determined using a BCA protein assay kit (Pierce, Thermo Scientific, Rockford, IL, USA).

Proteins (10–15 µg) were separated by SDS-PAGE (5–8% gradient, BioRad), transferred to nitrocellulose membranes and blocked for one hour at room temperature with 0.05% tween-phosphate buffer saline (PBST, Medicago, Uppsala, Sweden) with either 5% non-fat milk (BioRad) or BSA (Sigma). Membranes were incubated overnight with primary antibodies: anti-FKBP51 (1:1000 Abcam, ab126715) and anti-PPARG (1:1000, Cell Signaling, #2443). Anti-glyceraldehyde-3-phosphate dehydrogenase (GAPDH, 1:3000, Millipore, Temecula, CA, USA) was used as a loading control protein. Membranes were then washed with PBST buffer and incubated with appropriate horseradish peroxidase conjugated anti-rabbit (Cell Signalling Technologies) secondary antibody. Protein bands were then visualized using enhanced chemiluminescence with a high-resolution field and quantified with ChemiDoc^TM^ MP System (BioRad).

### Fluorescent staining of lipid droplets

On days 0, 7 and 14 of differentiation, the medium was removed and cells were washed with PBS and fixed with 4% formaldehyde (Histolab, Gothenburg, Sweden) for 15 minutes at room temperature. The cells were again washed two times with PBS and stained with a combination of 0.5 μg/ml of the fluorescent neutral lipid dye, BODIPY 493/503 (4,4-Difluoro-1,3,5,7,8-Pentamethyl-4-Bora-3a,4a-Diaza-s-Indacene; Molecular Probes, OR, USA) and 3.3 μM of the nucleic stain Hoechst 33342 in PBS. The cells were incubated with the dye mixture for 30 min, washed with PBS and imaged on the InCellis Fluorescent microscope (Bertin Instruments, Montigny-le-Bretonneux, France)

### Immunocytochemistry

To assess the expression of FKBP51 protein in the cells by immunocytochemistry, the same rabbit anti-FBKP51 antibody was used as for Western blot (Abcam; ab126715) (n = 4). At days 0, 7 and 14 of differentiation, the medium was removed, the wells were washed twice with PBS and subsequently incubated with 4% formaldehyde for 15 minutes at room temperature. The formaldehyde was then removed and the cells were washed three times with PBS and immediately incubated with the primary antibody solution (rabbit anti-FBKP51 antibody 1:500, 10% Normal Goat Serum (NGS, Sigma) and 0.1% Triton X-100 (Sigma) in PBS) overnight at 4 °C. The following day the cells were washed three times with PBS and incubated with the secondary antibody solution (Alexa Fluor® 594 goat anti-rabbit IgG (1:1000; Molecular Probes), 10% NGS and 0.1% Triton X-100 in PBS) for 2 hours at 4 °C. The cells were then washed three times with PBS. To quantify adipocyte differentiation, the cellular neutral lipids were also stained as well as nuclei as described above. Immunocytochemistry for assessment of PPARG protein levels was performed using a similar protocol but using the rabbit anti-PPARG antibody (Cell Signaling, #2443). Cells were blocked in 5% NGS and 0.3% Triton X-100 in PBS followed by incubation with the primary antibody (dilution 1:100 in 1% BSA/0.3% Triton X-100 in PBS) for 1 hour. Secondary antibody protocol and nuclei and lipid staining were the same as described for the FKBP51 antibody.

### Quantification of differentiation and immunofluorescence

All imaging was performed on the InCellis Fluorescent microscope using a DAPI Light Cube to visualize the nuclei, green fluorescent protein (GFP) Light Cube to visualize stained lipids with BODIPY, Texas-Red Light Cube to visualize the Alexa Fluor® 594 that stains FKBP51 and PPARG and as well as phase contrast for brightfield imaging.

Relative adipogenic differentiation was assessed at differentiation day 14 by quantification of the GFP signal from the BODIPY 493/503-stained neutral lipids, normalized to cell number from a minimum of 2 wells and 5 different randomly chosen images/well from a total of four independent experiments (10x magnification). The quantification was performed in batch mode using ImageJ scripts for fluorescent-based lipid droplet quantification and nuclei counting of the DAPI-signal from the Hoechst 33342 stain.

ImageJ was also used for quantification of FKBP51 and PPARG positive cells, where the cells with a TexasRed signal were counted in batch mode and calculated as a percentage of the total number of cells based on the Hoechst signal.

### Statistics

All data are shown as mean ± SEM unless otherwise indicated. All data were first checked for normality using the Shapiro-Wilk test, as well as analyzing histograms. A comparison between the mean of more than two groups was done using one-way ANOVA with repeated measures or mixed-effects model analysis as appropriate. The correction for the multiple comparisons was done by controlling the false discovery rate using original Banjamini and Hochberg method. A comparison between the two groups was done using a paired t-test or Wilcoxon test for normally and non-normally distributed data, respectively. P < 0.05 was considered to be statistically significant. All data were analyzed using GraphPad Prism version 8.

## Supplementary information

Supplementary Information.

## Data Availability

Data will be available upon request.

## References

[1] Kershaw EE, Flier JS (2004). Adipose tissue as an endocrine organ. J. Clin. Endocrinol. Metab..

[2] Lundback V (2018). FAM13A and POM121C are candidate genes for fasting insulin: functional follow-up analysis of a genome-wide association study. Diabetologia.

[3] Yan M, Li J (2019). The evolving CRISPR technology. Protein Cell.

[4] Claussnitzer M (2015). FTO Obesity Variant Circuitry and Adipocyte Browning in Humans. N. Engl. J. Med..

[5] Xue R (2015). Clonal analyses and gene profiling identify genetic biomarkers of the thermogenic potential of human brown and white preadipocytes. Nat. Med..

[6] Pereira MJ (2014). FKBP5 expression in human adipose tissue increases following dexamethasone exposure and is associated with insulin resistance. Metabolism.

[7] Menke A (2013). Genetic variation in FKBP5 associated with the extent of stress hormone dysregulation in major depression. Genes. Brain Behav..

[8] Hausl AS, Balsevich G, Gassen NC, Schmidt MV (2019). Focus on FKBP51: A molecular link between stress and metabolic disorders. Mol. Metab..

[9] Wochnik GM (2005). FK506-binding proteins 51 and 52 differentially regulate dynein interaction and nuclear translocation of the glucocorticoid receptor in mammalian cells. J. Biol. Chem..

[10] Wabitsch M (2001). Characterization of a human preadipocyte cell strain with high capacity for adipose differentiation. Int. J. Obes. Relat. Metab. Disord..

[11] Lee MJ, Fried SK (2014). Optimal protocol for the differentiation and metabolic analysis of human adipose stromal cells. Methods Enzymol..

[12] Chandrasekaran AP, Song M, Kim KS, Ramakrishna S (2018). Different Methods of Delivering CRISPR/Cas9 Into Cells. Prog. Mol. Biol. Transl. Sci..

[13] Yeh WH, Chiang H, Rees HA, Edge ASB, Liu DR (2018). In vivo base editing of post-mitotic sensory cells. Nat. Commun..

[14] Liang G, Zhang H, Lou D, Yu D (2016). Selection of highly efficient sgRNAs for CRISPR/Cas9-based plant genome editing. Sci. Rep..

[15] Sidibeh CO (2018). FKBP5 expression in human adipose tissue: potential role in glucose and lipid metabolism, adipogenesis and type 2 diabetes. Endocrine.

[16] Stechschulte LA (2014). FKBP51 reciprocally regulates GRalpha and PPARgamma activation via the Akt-p38 pathway. Mol. Endocrinol..

[17] Zhang L (2017). Loss of FKBP5 impedes adipocyte differentiation under both normoxia and hypoxic stress. Biochem. Biophys. Res. Commun..

[18] Stechschulte LA (2014). FKBP51 controls cellular adipogenesis through p38 kinase-mediated phosphorylation of GRalpha and PPARgamma. Mol. Endocrinol..

[19] Toneatto J (2013). Dynamic mitochondrial-nuclear redistribution of the immunophilin FKBP51 is regulated by the PKA signaling pathway to control gene expression during adipocyte differentiation. J. Cell Sci..

[20] Shao X (2016). Peroxisome Proliferator-Activated Receptor-gamma: Master Regulator of Adipogenesis and Obesity. Curr. Stem Cell Res. Ther..

[21] Denny WB, Valentine DL, Reynolds PD, Smith DF, Scammell JG (2000). Squirrel monkey immunophilin FKBP51 is a potent inhibitor of glucocorticoid receptor binding. Endocrinology.

[22] Balsevich G (2017). Stress-responsive FKBP51 regulates AKT2-AS160 signaling and metabolic function. Nat. Commun..

[23] Sidibeh CO (2017). Role of cannabinoid receptor 1 in human adipose tissue for lipolysis regulation and insulin resistance. Endocrine.

[24] Hahle, A., Merz, S., Meyners, C. & Hausch, F. The Many Faces of FKBP51. *Biomolecules* **9**, 10.3390/biom9010035 (2019). 10.3390/biom9010035 PMC6359276 30669684

[25] Stahn C, Buttgereit F (2008). Genomic and nongenomic effects of glucocorticoids. Nat. Clin. Pract. Rheumatol..

[26] Lundgren M (2008). Sex- and depot-specific lipolysis regulation in human adipocytes: interplay between adrenergic stimulation and glucocorticoids. Horm. Metab. Res..

[27] Kamble PG (2016). Lipocalin 2 produces insulin resistance and can be upregulated by glucocorticoids in human adipose tissue. Mol. Cell Endocrinol..

[28] Kamble PG (2018). Role of peroxisome proliferator-activated receptor gamma Pro12Ala polymorphism in human adipose tissue: assessment of adipogenesis and adipocyte glucose and lipid turnover. Adipocyte.

[29] Campenhout CV (2019). Guidelines for optimized gene knockout using CRISPR/Cas9. Biotechniques.

[30] Schmittgen TD, Livak KJ (2008). Analyzing real-time PCR data by the comparative C(T) method. Nat. Protoc..

