# Supplementary material for: Proof-of-concept for CRISPR/Cas9 gene editing in human preadipocytes: Deletion of FKBP5 and PPARG and effects on adipocyte differentiation and metabolism
Source: Sci Rep. 2020 Jun 29;10:10565. doi: 10.1038/s41598-020-67293-y (PMC7324390; doi:10.1038/s41598-020-67293-y)

**Supplementary material**

**Proof-of-concept for CRISPR-Cas9 gene editing in human preadipocytes: Deletion of FKBP5 and PPARG and effects on adipocyte differentiation and metabolism**

Prasad G Kamble^1^, Susanne Hetty^1^, Kristina Almby^1^, Casimiro Castillejo-López^2^, Xesús M Abalo^1^, Milica Vranic^1^, Maria J Pereira^1^, Jan W Eriksson^1^

^1^Department of Medical Sciences, Clinical Diabetology and Metabolism, Uppsala University, Sweden

^2^Department of Immunology, Genetics and Pathology.Science for Life Laboratory, Uppsala University

***Optimization of electroporation parameters for transfection of human preadipocytes***

Human adipose tissue-derived preadipocytes were electroporated with a Neon® Transfection system and The Neon® Transfection System 10 µL Kit (Thermo Fisher) as per the manufacturer's guidelines with slight modification. The electroporation parameters were optimized by transfecting cells with sgRNA targeting the human HPRT (Thermo Fisher, Cat no: A35524) locus and Cas9 protein (Thermo Fisher) using a combination of different pulse width and number of pulses given in table 1. Briefly, to form an RNP complex, chemically modified sgRNA (9.3 pmol/reaction) and TrueCut^TM^ Cas9 protein v2 (6 pmol/reaction) (both from Thermo Fisher) were mixed together in buffer R and incubated for 15 minutes at room temperature. In total 60000 cells were electroporated and transferred to a well in a 12 well plate in a . After 48 hours of transfection the cells were collected and genome-editing efficiency was tested by analyzing the locus-specific cleavage of genomic DNA using GeneArt® Genomic Cleavage Detection Kit (Thermo Fisher). The protocol was followed strictly in accordance with the manufactures instruction. The percent of insertions and deletions were calculated as suggested in the GCD kit protocol and shown in the figure 1. The primers sequence used for PCR amplification was Forward ACATCAGCAGCTGTTCTG and reverse GGCTGAAAGGAGAGAACT (purchased from Thermo Fisher).

**Table 1.** Different combinations of electroporation voltage, pulse width, and a number of pulses used for transfection of human adipose tissue-derived preadipocytes.

| Well number | Pulse Length (voltage) | Pulse Width (milliseconds) | Pulses |
| --- | --- | --- | --- |
| WT | Electroporated cells without sgRNA and Cas9. |  |  |
| S1 | 1700 | 20 | 1 |
| S2 | 1400 | 20 | 2 |
| S3 | 1750 | 20 | 1 |
| S4 | 1650 | 20 | 1 |
| S5 | 1350 | 20 | 2 |
| S6 | 1450 | 20 | 2 |
| S7 | 1350 | 20 | 2 |
| S8 | 1450 | 20 | 1 |
| S9 | 1500 | 20 | 1 |

**Figure 1**


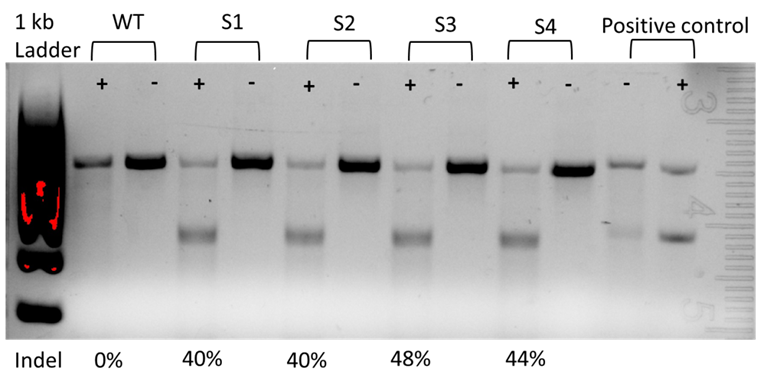

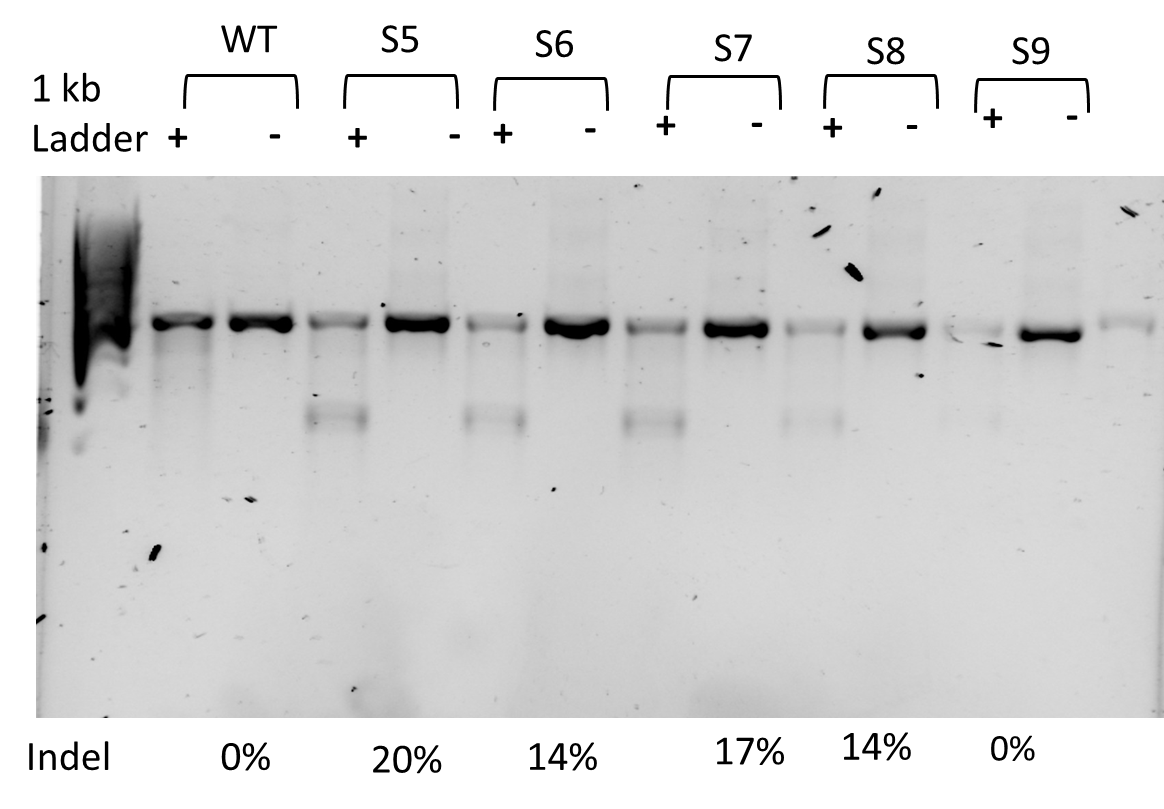


**Figure 1.** Gel images of genomic cleavage detection assay using human adipose tissue-derived preadipocytes transfected with sgRNA targeting HPRT locus and Cas9 protein. Different electroporation conditions are labeled on top of the gel (S1-S9) and the corresponding values are given in table 1. For each condition, cleavage efficiency (% insertion or deletion of nucleotide (indels) is shown at the bottom of the gel. WT (wild type) cells were only electroporated, without sgRNA and Cas9 protein.

**Western blot uncropped images:**

Figure 2c


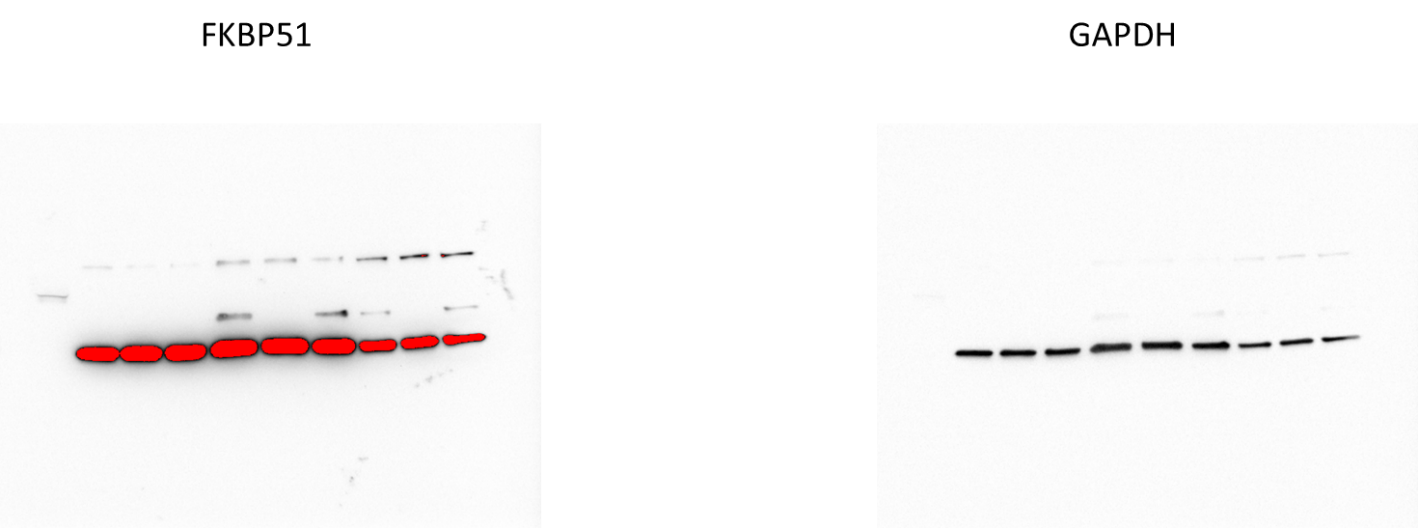


Figure 3b


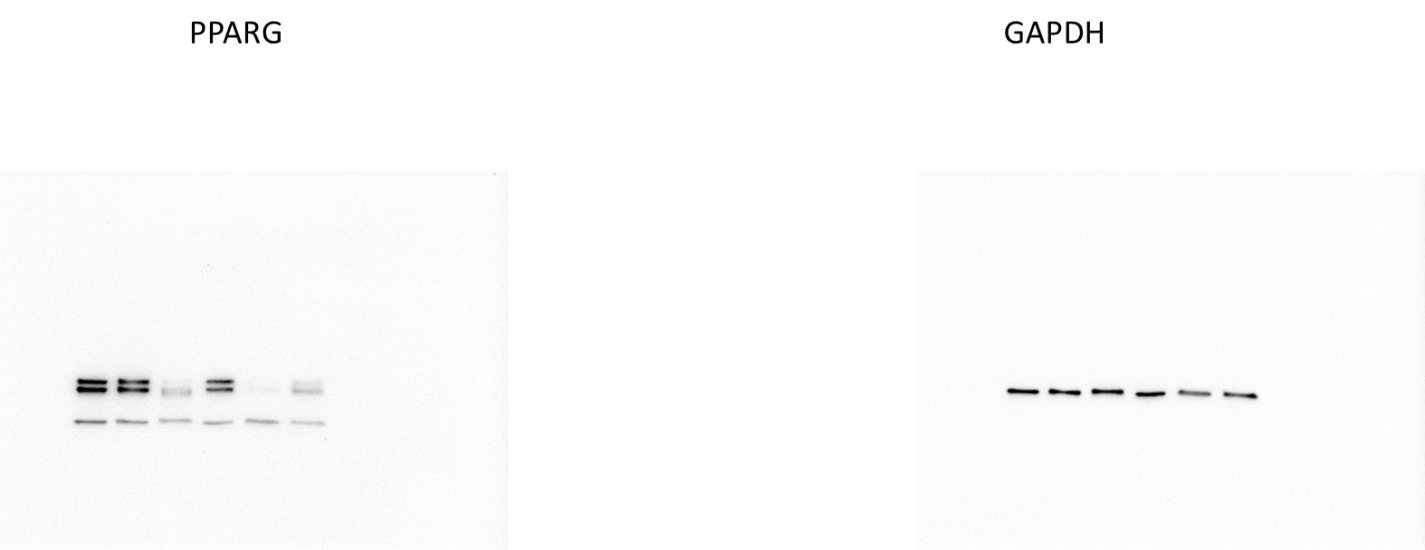

Supplement: Supplementary file 1 — Supplementary Information. [file 41598_2020_67293_MOESM1_ESM.docx]
